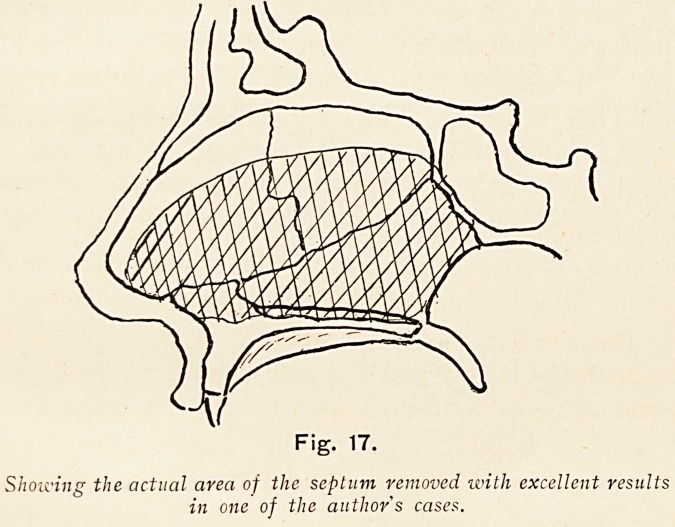# Operations for Deflections and Spurs of the Nasal Septum, with Special Reference to Sub-Mucous Resection

**Published:** 1907-03

**Authors:** P. Watson Williams

**Affiliations:** Laryngologist and Rhinologist, Bristol Royal Infirmary; Aural Surgeon, Deaf and Dumb Inst.; Lecturer on Diseases of the Nose and Throat, University College, Bristol.


### OPERATIONS FOR DEFLECTIONS AND SPURS OF THE NASAL SEPTUM, WITH SPECIAL REFERENCE TO SUB-MUCOUS RESECTION.

BY

P. WATSON WILLIAMS, M.D. Lond.,

*Laryngologist and Rhinologist, Bristol Royal Infirmary; Aural Surgeon, Deaf and Dumb Inst.; Lecturer on Diseases of the Nose and Throat, University College, Bristol.*

---

THE nasal septum is formed by the triangular cartilage, the perpendicular plate of the ethmoid, and the vomer, and is, therefore, divisible into the cartilaginous and the bony septum. These structures forming the septum may become fractured, dislocated, or deflected according to the nature of the injury or other primary cause of deformity. The cartilaginous septum is the most liable to dislocation or fracture, and most readily yields when subject to other causes of deflection mentioned below, and thus is most frequently the seat of defects requiring interference.

Deformities of the septum are very variable in shape, and it is convenient for clinical purposes to recognise three chief varieties. Deviations or deflections which are (1) C-shaped, as the septum may be bowed towards one side or the other, in an antero-posterior direction or in a vertical plane. (2) S-shaped where the bowing is in one direction anteriorly or below, and in the opposite direction posteriorly or above. These C- and S-shaped deflections are frequently due to or associated with dislocations of the cartilage from the vomer, and are frequently combined with the next class of ridges or thickenings along the line of such dislocation. (3) Outgrowths, spurs, crests, or spines. These are usually seen along the line of articulation of the triangular cartilage with the vomer, and may or may not be accompanied with actual dislocation of the cartilage. Fractures of the cartilage in a vertical direction due to traumatism, when the anterior margin of the cartilage is usually seen projecting into one or other nasal vestibule, are very frequently complicated by thickenings forming a ridge along the seat of the fracture, usually due to local perichondritis and thickening of the mucous membrane over the corresponding area. But such thickenings or ridges may arise without any deflection or dislocation of the cartilaginous or bony septum being obvious.

*Causes.*—In a very large number of cases no history of traumatism can be obtained. On the other hand, in a very large percentage where marked deviation or spurs are present, the history of a severe blow is obtainable, and leads to the conclusion that even in the absence of such history some forgotten blow or injury has been the determining cause, for it is obvious that very few pass their time of childhood without being exposed to causes which might give rise to such defects.

It is probable that in earlier life any deflections of the septum may be relatively slight in degree, symptoms only arising long after, when, either from irritation in the region of the resulting crest or from the gradual increase of the deflection due to the suction action of respiration, the original deformity has become aggravated. But considerable deformities and deflections of the septum often exist without any symptoms whatever, and it is certain that in at least a considerable number, when symptoms ultimately do arise, it is owing to conditions which have lead to turbinal hypertrophy.

The deformity of the palate resulting from nasal stenosis, viz., the V-shaped or vaulted palate is considered by some authorities to be a cause of septal deflections; while others consider that these septal deformities are due to the developing septum being out of proportion to the bony framework of the nasal fossæ. Probably in a certain proportion of cases these developmental factors influence the origin of the septal defects; but I am more and more convinced that traumatism is, even in the absence of the history of blows, by far the most frequent cause of the condition.

Fig. 1.

*Diagram showing the formation of the high narrow palate and the deformity of the nasal septum resulting from nasal obstruction. The arrows indicate the line of action of the compressing force.* (*After* LAMBERT LACK.)

Inasmuch as many persons who complain of no symptoms and exhibit no signs of nasal defects of any kind whatever are found in the course of examination to have considerable septal deflections or crests, it is important in determining the necessity for interference to be guided by the presence or absence of symptoms which call for removal of the septal defect. Yet very distressing symptoms may arise in cases where the nasal passages are sufficiently patent to allow nasal respiration from the lower portion of the nasal passages, but where, either owing to organic obstruction or to persistent nasal catarrh, the normal air tract is so stenosed as to prevent respiration along that tract.

Investigations such as those of Scheff and Kayser, and of

Parker, have shown that the inspired air normally ascends in a curved direction from the vestibule into the middle and superior

Fig. 2.

*Diagram to show the normal path of inspired air through the nasal passages.*

meatus, and thence gradually descends posteriorly to the choanæ. (Fig. 2.) Stuffiness in the nose resulting from ordinary cold, and the deficient respiration shown by children who are subject to adenoid growths, is essentially due not so much to actual stenosis as to the rhinitis and catarrh which prevents respiration through these normal air tracts. So we often find in cases of septal deflection that patients exhibit symptoms of nasal stenosis when the deflection is somewhat high up and far back, while the combined respiratory capacity of the lower nasal passages would suffice for respiration. The observance of this fact becomes all the more important when one has to choose the particular form of operation for the relief of a given case,

Fig. 3.

*Double or S-shaped septal deflection —rectified by sub-mucous resection, and by ablation of the overfull anterior end of the left inferior turbinal.*—W. W.

since we shall see that for this latter class the only satisfactory method is that of submucous resection. (Fig. 3.)

Undue fulness of one or more of the turbinated bodies, amounting to hypertrophy, may cause nasal stenosis, and it is sometimes necessary to reduce the enlarged turbinal by the galvano-cautery or by partial ablation. When the septum is deflected, the turbinated body corresponding to the resulting concavity very frequently undergoes a compensatory hypertrophy, so as to project into and partly fill this concavity. In some cases the nasal stenosis may be overcome from simple reduction of the size of the turbinals, by removing quite a small portion, rendering any interference with the septum unnecessary. But when a septum has to be straightened, such hypertrophic enlargement of the turbinal on the concave side will obviously tend to block the air-way more than ever, so that the previously patent side would become stenosed. This point must always be taken into consideration before rectifying the septal deformity, the anterior end of the hypertrophied turbinal, whether it be the middle or inferior, being ablated or otherwise reduced either a short time before or at the same time as the septal operation.

*Operative Methods.*—Of the numerous methods that have been advocated for the restoration of the septum, it is safe to say that many will now be relegated to the past in view of the eminently satisfactory results which can be obtained by submucous resection. Space prevents my alluding to more than three of the chief methods now employed, viz. Gleason's operation, Moure's operation, and sub-mucous resection, the latter alone calling for detailed description on account of its applicability to all cases and of the technical difficulties in its performance.

**Gleason's operation** can be commended where one has to deal with fairly hollow C-shaped deflections of the septum over a limited area, restricted to the triangular cartilage, and where there is no marked thickening of the septum as a whole.

It consists in making a U-shaped flap of the deviation either by transfixing the deviated portion of the convex side by a narrow-bladed knife, which is passed through the cartilage just in front of the higher portion of the deflection, and then made to reappear on the same side by transfixing the cartilage again just posteriorly to the deflected portion, the knife being then carried vertically downwards until it is below the deflection, when it reappears; or a saw is made to cut the deflection from below upwards with much the same result. In either case the tongue-like flap of septum, with its mucous membrane and perichondrium intact, is hanging attached by its superior border.

Fig. 4.

*To show the method of making the flap in Gleason's operation.*—W. W.

This is then forcibly pressed through to the concave side with the finger, care being taken to overcome the resiliency of the cartilage at its attachment. (Fig. 5.) The oblique direction of the incision ensures that the margins of the flap extend somewhat beyond the margins of the septal incision: thus when the flap has been passed through it cannot spring back again. If necessary, a splint is inserted on the concave side, sufficient to maintain slight pressure on the margins of the flap against the corresponding portions of the septum on the concave side, and this in the course of a few days results in union. In the majority of cases which are suitable for the operation the result is very satisfactory, but it is obvious that if the septum is thickened where it was deflected this

Fig. 5.

*One method of pushing the U-shaped flap in the septum to the concave side.*

Fig. 6.

*Diagram showing how the flap is automatically retained in position.*

thickening will be liable to cause obstruction of the formerly patent side. (Fig. 6.)

**Moure's operation** consists in making the incision from before backwards, along the horizontal crest or lower portion of the

Fig. 7.

*Showing the lines of incision in Moure's operation.*

septal deviation parallel to the floor of the nose, and a second incision parallel to the anterior margin of the triangular cartilage, along the whole length of the deflection above, and with the finger or suitable septum forceps causing fracture of the cartilage towards the formerly unobstructed side, so as to overcome the resiliency of the cartilage. The cartilage is kept in the new position by means of Moure's or other suitable nasal plugs, and these at the end of a week are dispensed with, when the union has generally taken place. In some cases it may be necessary to subsequently trim the margins. Here again it is obvious that the operation can only be applicable to cases where there is sufficient room on the non-obstructed side to receive the deflected portion, and this can only be where deflection is simple, and has not undergone much thickening. (Fig. 7.)

The advantage of these operations is that they are quickly performed, and do not call for such technical skill as is essential for successful sub-mucous resection.

**Sub-mucous resection** essentially consists in the removal of the deviated portion of the cartilaginous and bony septum, while at the same time completely preserving the mucous membrane and perichondrium, a thickened and deflected septum being replaced by one that is thin, straight and stiff. It is thus suitable for every kind of septal deflection or spur, whether it is or is not associated with thickening of the septum, while for cases where the septum is considerably thickened it is the one method which most satisfactorily overcomes the difficulty, and ensures normal and patent nasal passages without destruction of the mucous membrane.

It is always possible in persons of good nerve and considerable self-control to do this operation with local anæsthesia alone, but the long time often required to do all that is necessary makes it very trying to the majority of individuals; and the prolonged strain, even in the absence of pain, makes it preferable to resort to general anæsthesia as a rule. If one depends on local anæsthetics, cocaine, eucaine, or novocaine can be used, and must be applied in solutions of considerable strength; but the fact that cocaine is not infrequently trying to the patient makes it the more desirable to have them under general anæsthetics whenever possible. In any case, adrenalin solution should be applied, so as to cause vascular constriction, and it is important to allow sufficient time for the action of the adrenalin to take place before commencing the operation.

Difficulties attending local anæsthesia for septal resection would seem to have been satisfactorily met by Miller's method of locally applying a solution made by placing 20 grains of cocaine crystals in a shallow dish, and dropping sufficient adrenalin chloride solution 1 to 1,000, to dissolve the crystals. The solution is carefully applied to the area of mucosæ to be operated on. To better the patient's self-control, he administers a draught just before applying the cocaine, containing 10 grains each of the bromides of sodium, potassium and ammonium, with 1 drachm of aromatic spirit of ammonia. Miller reports that his last forty cases were operated on with this method of local anæsthesia painlessly, and with minimum of hemorrhage.

The patient should be lying on the back, with head and shoulders raised, and a very good illumination is essential.

The incision for simple ridges and spurs should extend from behind forwards along the summit of the ridge in its whole length, turning upwards for a quarter of an inch at the anterior extremity, the subsequent stages of the operation being similar to that for general deflection.

There are three different methods of incising the mucous membrane :—(1) The triangular ⅃-shaped incision ; (2) the single buttonhole incision ; (3) the author's method of incising the mucous membrane on both sides.

**⅃-shaped incision.**—If the variety of deflection is double-angled, with a vertical and horizontal crest, as shown in the accompanying figure, the first incision is usually made as suggested by Freer, along the angle of vertical deflection, beginning high up above the deflection, and extending right down to the horizontal ridge. Then a horizontal incision is made along the crest of the ridge, extending from the bottom of the vertical cut forwards almost to the front of the septum. This incision should cut just into but not through the cartilage, for if the muco-perichondrium be not divided, when one comes to lift the muco-perichondrium the mucous membrane alone may be separated and raised from the perichondrium beneath, instead of both being together raised from the cartilage. A triangular, anterior flap of muco-perichondrium is thus outlined, and this

Fig. 8.

*The ⅃-shaped incision, the mucosa being raised towards the front, exposing a triangular piece of the cartilage, which is cut through along the dotted line corresponding with the base of the exposed triangle.*

should be carefully reflected, and then held forward by a small pledget of wool, much care being taken to avoid perforation of this anterior flap. The muco-perichondrium is then raised below the horizontal incision by means of a suitable elevator right down to the floor of the nose. Next the perichondrium of the septum posterior to the vertical incision is lifted until the whole has been removed corresponding to the septal deflection, extending down to the floor of the nose, and if necessary to the posterior border of the vomer. In this way the cartilage, and where necessary the bony septum, is bared and exposed on the convex side over and somewhat beyond the whole area of deflection. Either with a round-edged chisel or a suitable septum knife, the cartilage is then incised, the incision extending *along the base* of the triangular flap, care being taken to leave at least a quarter of an inch corresponding to the anterior free border of the septum above, in order that there may be no risk of the falling-in of the nose. The incision must not extend through the perichondrium on the opposite side, and in making this incision the left forefinger should be inserted into the opposite nostril, so that no puncture shall be made. The muco-perichondrium is then raised from the concave side over the area corresponding to that alluded to in the first instance, care being taken to make the reflection right down to the floor of the nose on this side too.

**The single buttonhole incision** may be made about a centimetre and a half behind the septum cutaneum or columella, near the floor and extending upwards and forwards, being about threequarters of an inch long, nearly parallel to the anterior free margin of the cartilage but curving away from it below. The muco-perichondrium is then lifted on the convex side as in the first instance, but without making any triangular anterior flap. The cartilage is next incised without cutting through or perforating the mucosa on the opposite side, and the muco-perichondrium lifted from the concave side from before backwards.

**The author's method of incision on both sides.**—In many cases it is a matter of difficulty to incise the triangular cartilage in the manner described above without perforating the perichondrium, which is lying intact with it; and in order to avoid this contingency I have been in the habit of first making a small incision of the mucous membrane on the concave side, *well in front* of the site selected for the usual buttonhole incision, which is to be made on the convex side. A very narrow elevator is inserted so as to raise the muco-perichondrium, and by a movement of the distal end of this elevator upwards and downwards the muco-perichondrium is lifted from a considerable area on the concave side. (Figs. 9 and 12.) The elevator is then drawn out through the initial puncture, much as one would use a tenotomy knife. In this way the muco-perichondrium on the concave side, having already been lifted, when the incision comes to be made

Fig. 9.

*To show the author's method of making a small anterior incision on the concave side.*

Fig. 10.

*The usual buttonhole incision on the convex side. The dotted line in front shows the position of the small anterior incision on the concave side.*

in the usual way through the cartilage from the convex side, there should be no risk of perforation, because the curtain of muco-perichondrium on the concave side is simply pushed in

Fig. 11.

*Narrow perichondrium reflector for use on the concave side.*

front of the knife. The subsequent stages of operation are the same, whatever incision has been made. (Fig. 10.)

Speaking generally, the advantage of the L-shaped incision is that the incisions are made along the crests or angles of the deflections, and, as Freer has pointed out, it is easier to dissect the

Fig. 12.

Fig. 13.

*The two curtains of muco-perichondrium held apart, exposing the cartilage.*

muco-perichondrium from the summit of the ridge downwards on either side than it is by incising altogether in front of it to dissect the perichondrium first up to and along the summit and downwards along the farther side. Especially is this an advantage if the vertical ridge be sufficiently near the front to be accessible. When, however, the deflections are situated well back it is a greater advantage to utilise the buttonhole incision, or, to my mind, the double buttonhole which I have described.

Having thus laid bare the entire area of the septum, both cartilaginous and bony, corresponding to the deflections which may extend only to the quadrilateral cartilage, or, as we have seen, occupy the vomer and perpendicular plate of the ethmoid it finally remains essential to remove the whole of this deflected area. If any portion of the deflected area be left above or below, although it may seem insignificant as the cause of subsequent stenosis, it becomes of importance owing to its preventing the two curtains of muco-perichondrium (Fig. 13) hanging vertically in apposition in the mid-line, therefore interfering with their subsequent adhesion, and also because any intervening space may become filled with blood-clot, which may suppurate or become filled up with granulation tissue, leaving a thickened or irregular septum, instead of a thin, straight septum, which is the great end of the operation. In removing the deflection, it is convenient to hold apart the two curtains of muco-perichondrium by means of a long speculum, such as St. Clair Thomson's (Fig. 14) or Tilley's, or one may use Killian's speculum for median rhinoscopy. The cartilage should be removed by Ballenger's swivel knife (Fig. 15), applying it either at the lowermost part of the cartilaginous margin, or the uppermost angle, carrying it forwards until it reaches the bony septum, being then turned upwards or downwards as the case may be, and encircling if necessary the whole of the cartilage between the maxillary crest to within a quarter of an inch of the superior free margin of the cartilage. The knife is drawn out, having cut through the cartilage, which can then be lifted out readily with forceps. Subsequently the perpendicular plate of the ethmoid or vomer, if the

Fig. 14.

*St. Clair Thomson's speculum.*

seat of deflections or crests, are clipped away with cutting forceps.

The maxillary nasal spine and vomerine ridge must now be dealt with. The nasal spine may be prised away with forceps or removed by hammer and chisel, great care being taken not to wound the lower portions of the perichondrial flaps. If the ridge posteriorly is displaced or thickened it is clipped away, and then it only remains to wash away the debris, bring the curtains of muco-perichondrium into position, and very lightly pack the nasal passages on either side with strips of gauze, just sufficient to exert very slight pressure. By these means we keep the two flaps in their position, and not only ensure primary union, but prevent the accumulation of blood between the layers, which may cause subsequent trouble by suppurating. The following day all packing may be removed and the nose cleansed with warm antiseptic and alkaline solutions, which may be repeated daily

Fig. 15.

*Ballenger's swivel knife, modified to hold flaps of mucosa apart on cutting the cartilage.*

Fig. 16.

*Wood's forceps for removal of maxillary crest.*

until at the end of a week the parts will be healed and complete union have taken place.

The drawback to the operation, which has such eminently satisfactory results, is that it takes considerable time, and though much care and patience be observed, perforation may result. Although these, if far back, may be of no moment, yet when they are near the anterior end of the septum they are found to catch dust or form crusts, or if they are only small perforations they may cause whistling sounds during respiration through the nose. Such contingencies, however, would rarely happen in the hands of a skilful operator.

Occasionally a septum becomes not only enormously deflected, but in process of development obviously increases out of all proportion to the other nasal structures. In one case under my care the deflection was so pronounced that the angle caused a slight elevation on the outside of the nose, and extended right across the nasal passage so as to completely obtrude it, while the posterior deflection on the opposite side also caused stenosis there. The intervening area on the concave side showed an enormous depression, which at first sight looked like a very large perforation. The case also was complicated by double empyema of the antra, doubtless mainly resulting from the retention of the nasal secretion owing to the deflections. In this case it was quite impossible to do resection, and the only way to relieve it was to resect a portion of the septum and leave the perforation.

It is remarkable that despite the entire removal of the cartilage between the layers of the perichondrium, which for a time will move like a curtain when touched with a probe, or even during respiration, yet nevertheless will become so stiff in the course of a few months as to give rise to the impression that cartilage has re-formed, which, of course, never can occur.

A very large number of these operations have now been done over a period of several years, and there seems to be no risk of depression of the nose externally. But in order to render this impossible, it is desirable to leave a quarter of an inch of free margin corresponding to the upper border of triangular cartilage above the lateral cartilage. Cases have been recorded where even a severe blow on the nose in patients who have undergone sub-mucous resection no deflection or depression has resulted.

How far one can wisely remove the bony or cartilaginous septum in children it is hardly safe to say without a larger number of cases before us than have yet been reported, but several instances in which quite young children have undergone the operation successfully, and without interference with their nasal development, have been recorded, and I have myself in one case removed a large portion of the anterior end of the triangular cartilage in a boy aged nine, and although that was

Fig. 17.

*Showing the actual area of the septum removed with excellent results in one of the author's cases.*

several years ago and he is grown up, his nose has developed well without trace of depression.

Indeed, it may be said that in children where stenosis exists nasal development would be far more interfered with if the cause be left, apart from all the other unfortunate results to nasal stenosis in a growing child, than could be the case from an adequate removal of the septal defects.

The Krieg-Bonninghaus operation, known also as the **Fenster resection,** consists in removing the whole of the cartilage or bone forming the deflection, together with the corresponding part of the muco-perichondrium on the convex side, and leaving the single bared muco-perichondrium of the concave side to form the new septum. It was originally introduced by Krieg and revived by Bonninghaus in 1900, but though still preferred by some operators, has been generally superseded by the more satisfactory, though slightly more difficult, operation of sub-mucous resection. An ⌋-shaped incision is made on the convex side, the vertical incision in front of, and the horizontal extending below the whole of the deflection. The incision is made to cut through the cartilage without perforating the mucosa of the concave side, which is then raised as in the sub-mucous operation. The deflection, together with the mucous membrane of the concave side, is then removed bodily by scissors or cutting forceps. The fact that this procedure leaves a large bare surface of the muco-perichondrium of the other side, which takes some weeks to granulate and become covered with epithelium, is a serious drawback, though the ultimate result is usually satisfactory. The operation is nearly as difficult and tedious as the sub-mucous method, but the after-treatment is much more tedious, and the result is not so certain to be satisfactory, and I now never have recourse to it.

REFERENCES.

**Bonninghaus**—*Arch. f. Laryngol.*, ix. 269.
**Freer**—*J. Am. M. Ass.*, 1902, xxxviii. 636; 1903, xli. 1391; *Tr. Am. Laryngol. Assoc.*, 1905, p. 29.
**Hurd**—*Med. Rec.*, 1905, lxviii. 853.
**Killian**—*Verhandl. d. Gesellsch. d. Nat. u. Aerzt*, 1899.
**Kreig**—*Berl. klin. Wchnschr.*, 1899, xxvi. 699, 719.
**Lack**—*Diseases of the Nose*, 1906, pp. 65, 104 *et seq.*
**Miller**—*Med. Rec.*, No. 8, lxxi. 311.
**Moure**—*J. Laryngol.*, 1901, xvi. 163.
**Parker**—*Ibid.*, 1901, xvi. 345.
**Scheff and Kayser**—*Ibid.*, 1895, ix. 64.
**St. Clair Thomson**—*Med.-Chir. Trans.*, 1906, lxxxix. 655.

## Figures and Tables

**Fig. 1. f1:**
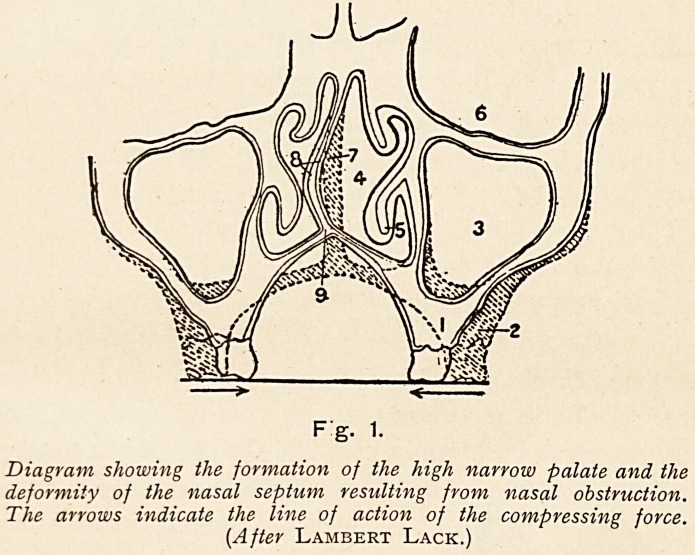


**Fig. 2. f2:**
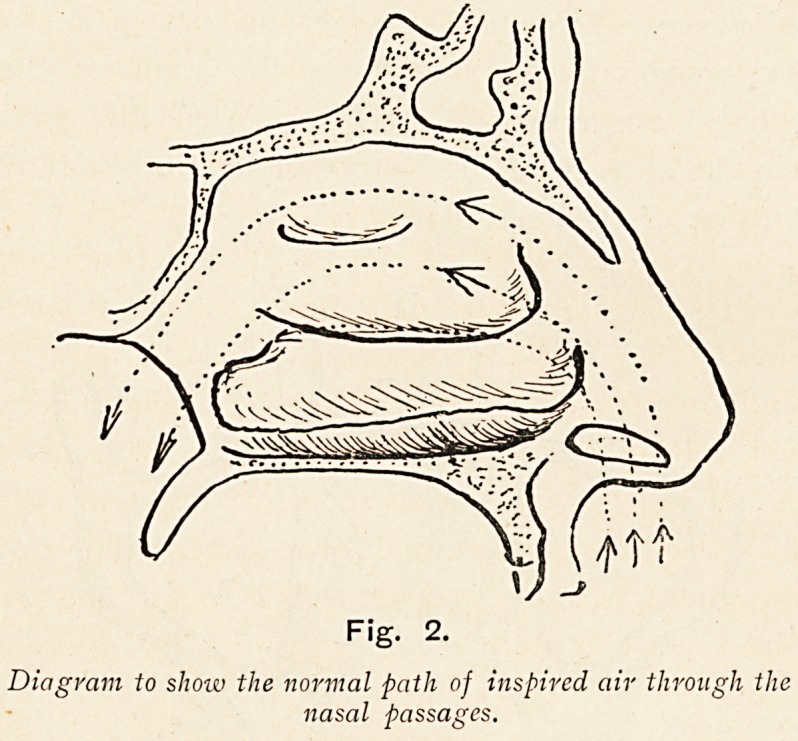


**Fig. 3. f3:**
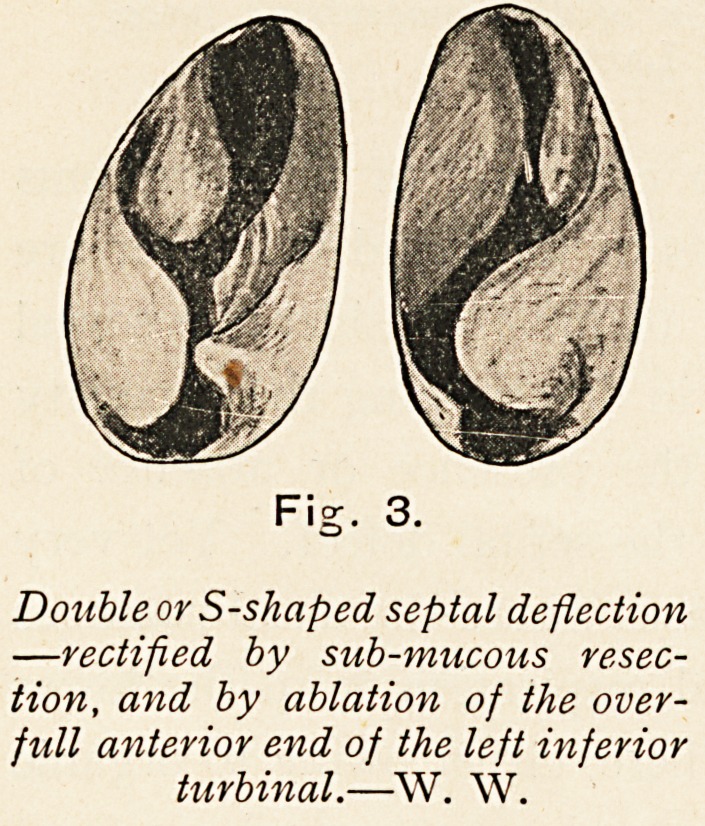


**Fig. 4. f4:**
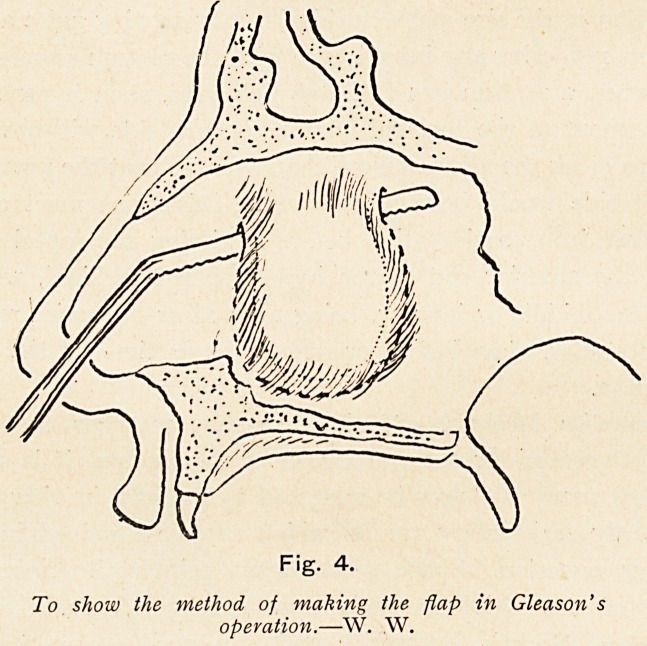


**Fig. 5. f5:**
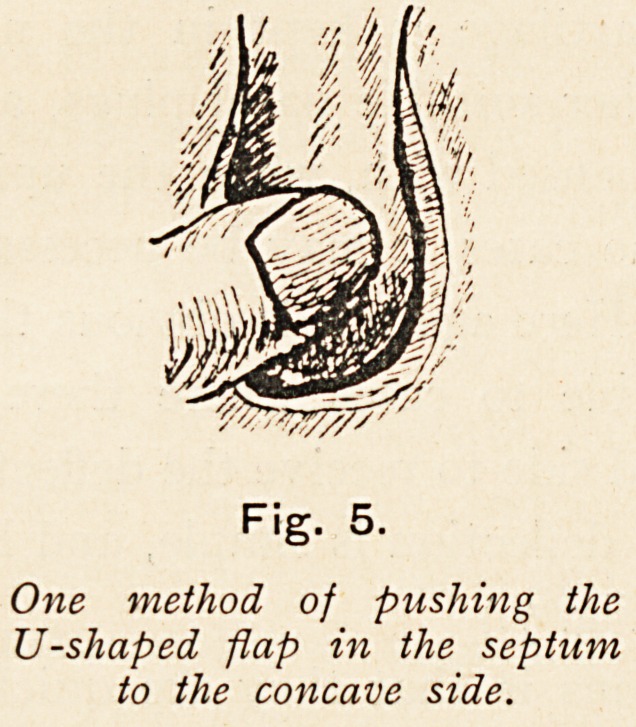


**Fig. 6. f6:**
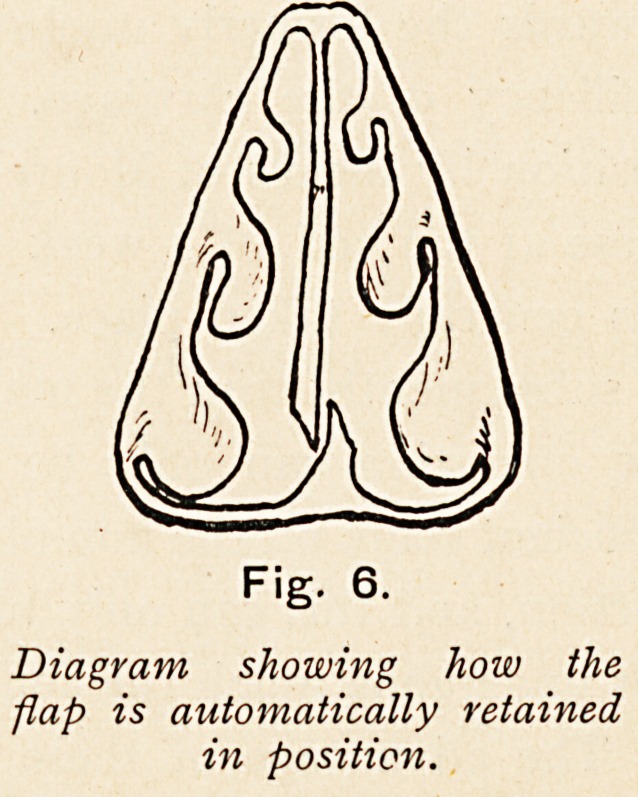


**Fig. 7. f7:**
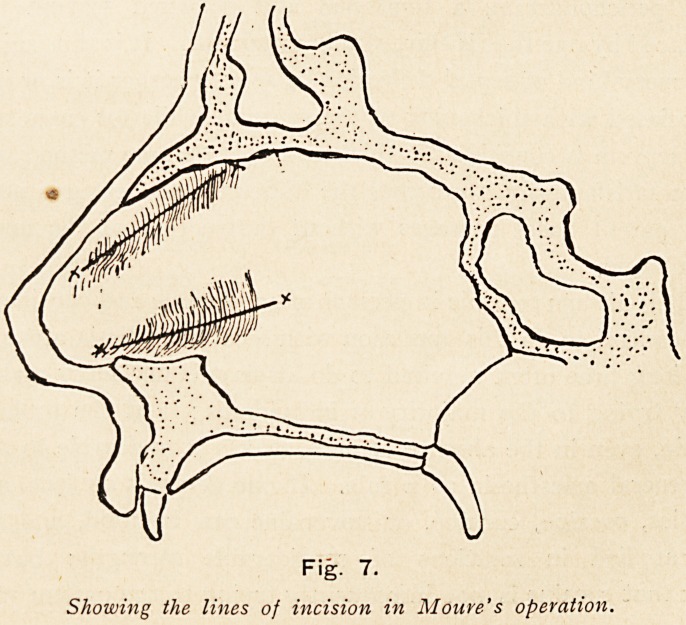


**Fig. 8. f8:**
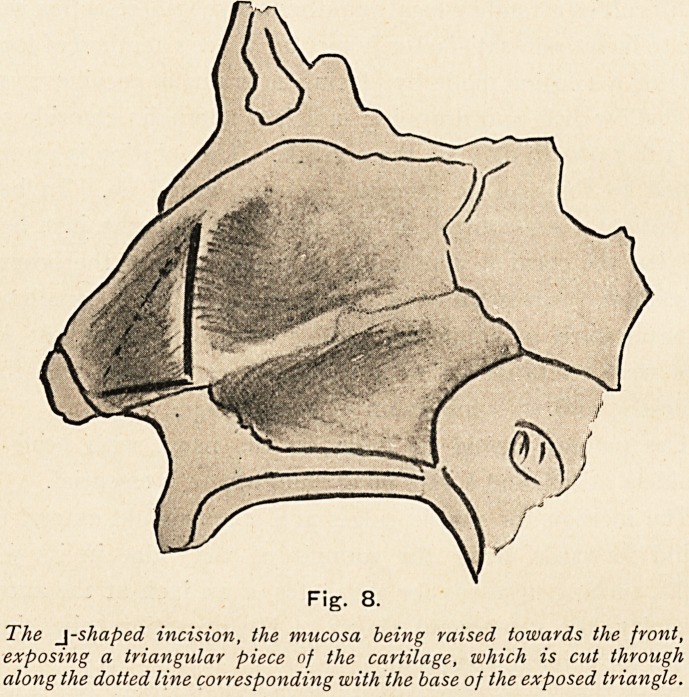


**Fig. 9. f9:**
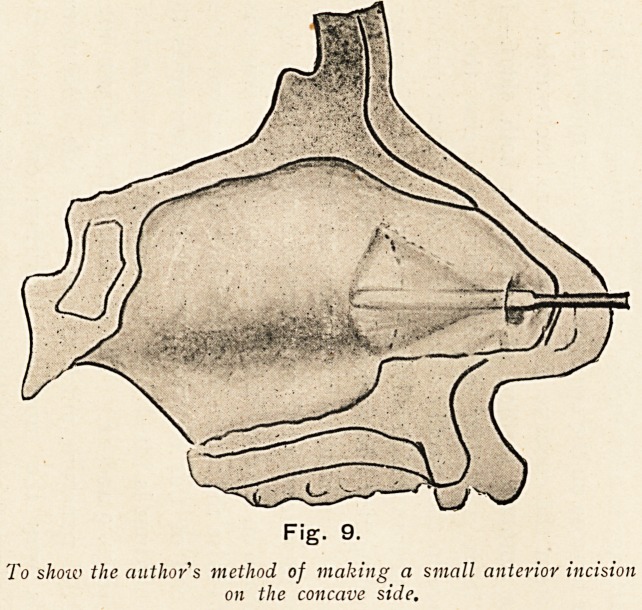


**Fig. 10. f10:**
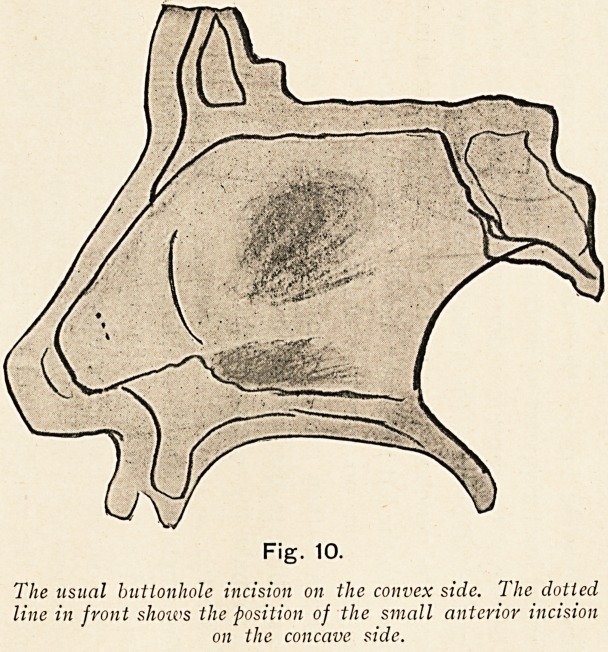


**Fig. 11. f11:**
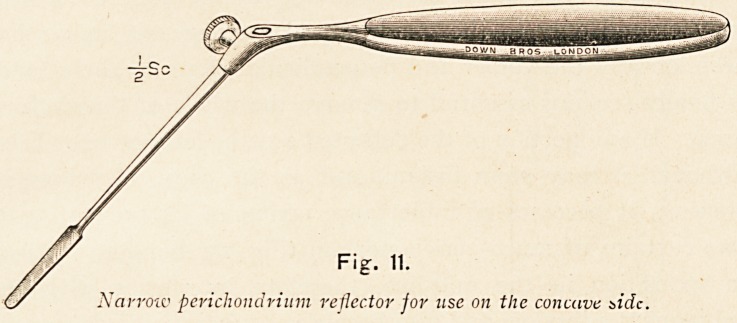


**Fig. 12. f12:**
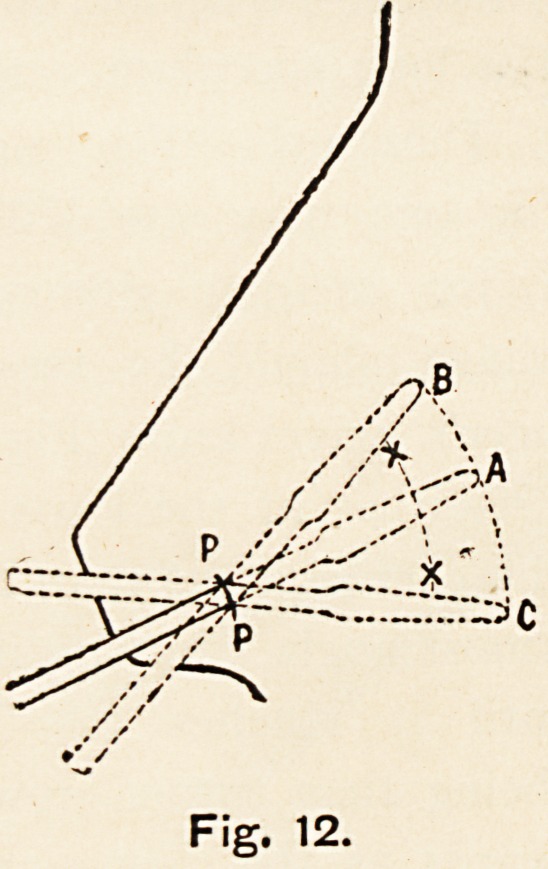


**Fig. 13. f13:**
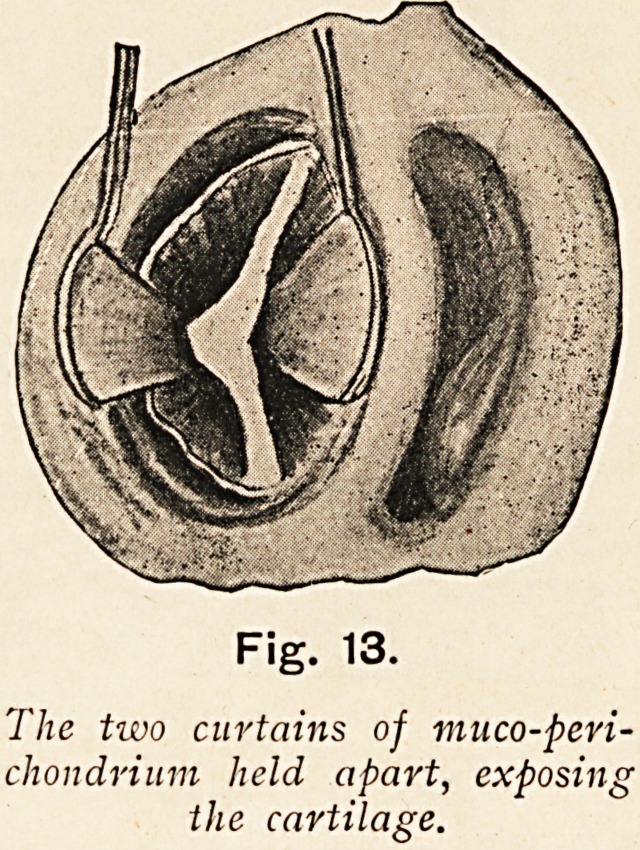


**Fig. 14. f14:**
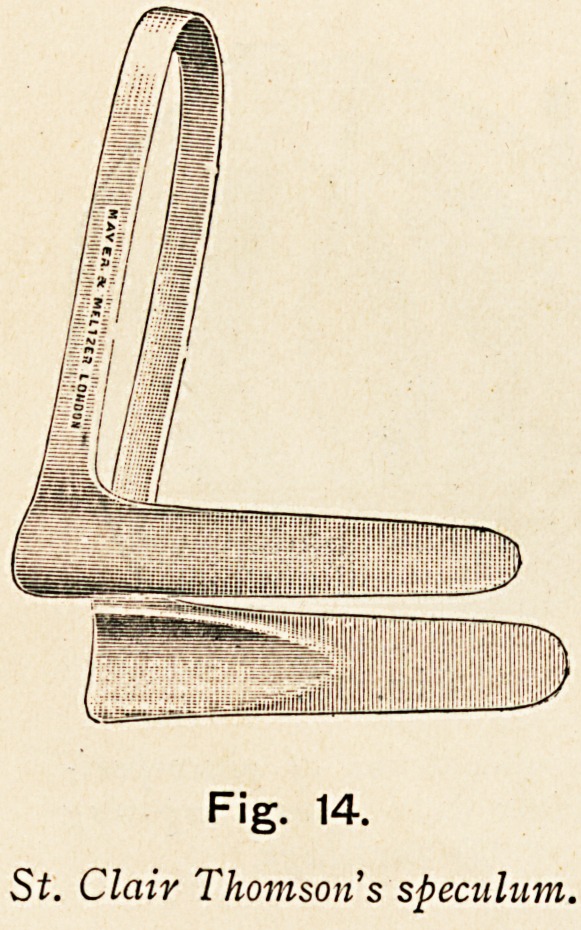


**Fig. 15. f15:**
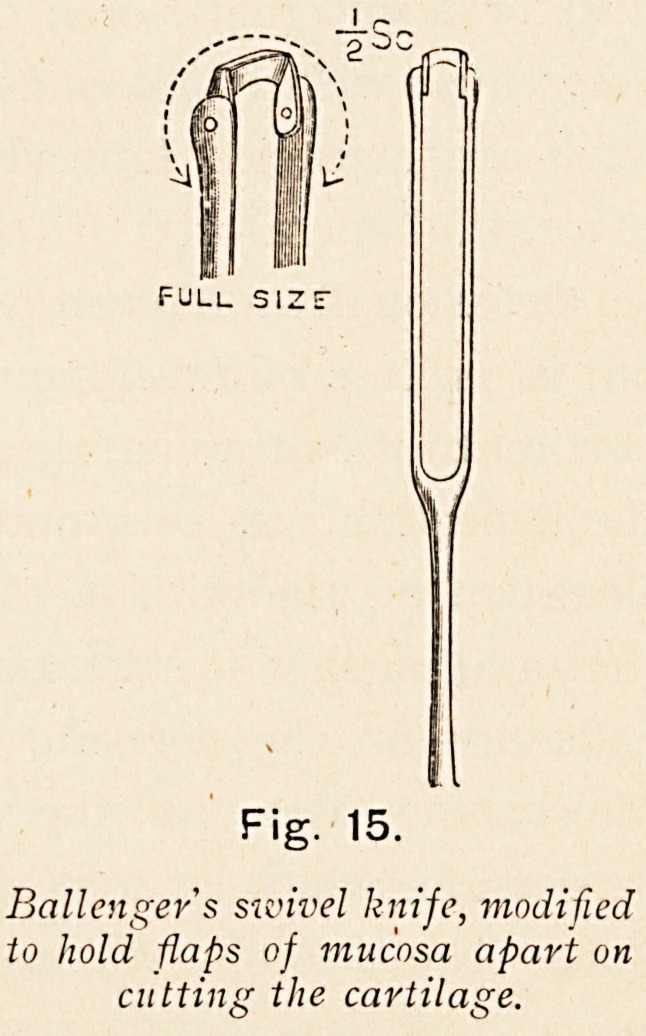


**Fig. 16. f16:**
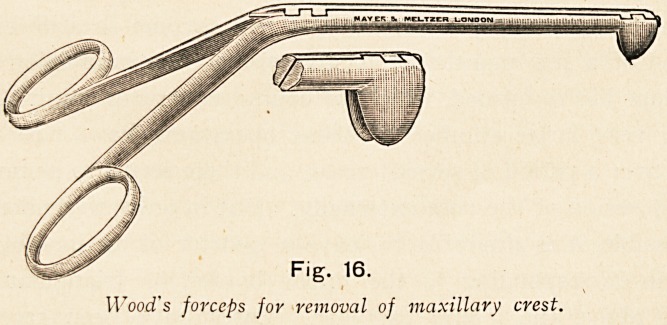


**Fig. 17. f17:**